# Comparative genomic analysis of *Halomonas campaniensis* wild-type and ultraviolet radiation-mutated strains reveal genomic differences associated with increased ectoine production

**DOI:** 10.1007/s10123-023-00356-y

**Published:** 2023-04-17

**Authors:** Zhibo Wang, Yongzhen Li, Xiang Gao, Jiangwa Xing, Rong Wang, Derui Zhu, Guoping Shen

**Affiliations:** Research Center of Basic Medical Science, Medical College of Qinghai University, Xining, 810016 China

**Keywords:** Ectoine, *Halomonas*, UV mutagenesis, Ectoine producer, Ectoine yield, Genomic analysis

## Abstract

Ectoine is a natural amino acid derivative and one of the most widely used compatible solutes produced by *Halomonas* species that affects both cellular growth and osmotic equilibrium. The positive effects of UV mutagenesis on both biomass and ectoine content production in ectoine-producing strains have yet to be reported. In this study, the wild-type *H. campaniensis* strain XH26 (CCTCC^M^2019776) was subjected to UV mutagenesis to increase ectoine production. Eight rounds of mutagenesis were used to generate mutated XH26 strains with different UV-irradiation exposure times. Ectoine extract concentrations were then evaluated among all strains using high-performance liquid chromatography analysis, alongside whole genome sequencing with the PacBio RS II platform and comparison of the wild-type strain XH26 and the mutant strain G_8_-52 genomes. The mutant strain G_8_-52 (CCTCC^M^2019777) exhibited the highest cell growth rate and ectoine yields among mutated strains in comparison with strain XH26. Further, ectoine levels in the aforementioned strain significantly increased to 1.51 ± 0.01 g L^−1^ (0.65 g g^−1^ of cell dry weight), representing a twofold increase compared to wild-type cells (0.51 ± 0.01 g L^−1^) when grown in culture medium for ectoine accumulation. Concomitantly, electron microscopy revealed that mutated strain G_8_-52 cells were obviously shorter than wild-type strain XH26 cells. Moreover, strain G_8_-52 produced a relatively stable ectoine yield (1.50 g L^−1^) after 40 days of continuous subculture. Comparative genomics analysis suggested that strain XH26 harbored 24 mutations, including 10 nucleotide insertions, 10 nucleotide deletions, and unique single nucleotide polymorphisms. Notably, the genes *orf00723* and *orf02403* (*lipA*) of the wild-type strain mutated to *davT* and *gabD* in strain G_8_-52 that encoded for 4-aminobutyrate-2-oxoglutarate transaminase and NAD-dependent succinate-semialdehyde dehydrogenase, respectively. Consequently, these genes may be involved in increased ectoine yields. These results suggest that continuous multiple rounds of UV mutation represent a successful strategy for increasing ectoine production, and that the mutant strain G_8_-52 is suitable for large-scale fermentation applications.

**Supplementary Information:**

The online version contains supplementary material available at 10.1007/s10123-023-00356-y.

## Introduction

Gram-negative *Halomonas* species are halophilic bacteria that live in saline or hypersaline environments (Hadibarata et al. 2023; Opara et al. 2023). The *Halomonas* genus contains the most moderately halophilic species in the family *Halomonadaceae* that comprises 102 identified species (http://www.bacterio.net/halomonas.html). *Halomonas* are well-known producers of organic compatible solutes and strains of the genus produce ectoine or 5-hydroxyectoine to facilitate osmotic equilibrium of the cytoplasm with surrounding environments (Czech et al. 2018; Fatollahi et al. 2021; Zhao et al. 2022). Ectoine is synthesized via a pathway comprising enzymes encoded by the three genes *ectA*, *ectB*, and *ectC* (Widderich et al. 2016; Dutta and Bandopadhyay 2022). Several *Halomonas* species including *H. ventosae* (Zhu et al. 2021), *H. elongata* (Pfeiffer et al. 2017; Zhang et al. 2022a, b, c), *H. boliviensis* (Gagliano et al. 2022; Sushmitha et al. 2022), *H. salina* (Dong et al. 2023; Gadallah et al. 2023), and *H. hydrothermalis* (Zhao et al. 2022) have been shown to synthesize ectoine. Concomitantly, previous studies have shown that ectoine has a wide range of applications in the biochemical, medical, cosmetic, and skin care fields, in addition to its potential role as a therapeutic agent for certain diseases (Pérez et al. 2021; Tuesta-Popolizio et al. 2021). Thus, a demand currently exists for the mass production of ectoine.

Industrial ectoine production is currently facilitated by several different large-scale fermentation strategies including batch, fed-batch, repeated fed-batch, combined two-step fed-batch, continuous, and bacterial milking fermentation processes (Lang et al. 2011; Vandrich et al. 2020; Dong et al. 2021, 2023; Fatollahi et al. 2021; Zhang et al. 2022a, b, c). Ectoine production yields from fermentation primarily depend on the salt concentration of the medium, carbon, and nitrogen ratios involved in metabolic overflow, strain growth rates, and cellular densities. Moreover, these factors are affected by culture conditions including temperature and pH (León et al. 2018; Piubeli et al. 2018; Weinisch et al. 2018; Dong et al. 2021; Jiang et al. 2022). Optimal ectoine-producing strains have been suggested to require rapid cellular proliferation (e.g., an OD_600_ value of approximately 1.2 after 8 h), wide salinity tolerance ranges (e.g., 0–3.0 M NaCl), and maximal extracellular ectoine release rates. Nevertheless, many strains isolated from high-salt environments that exhibit reduced ectoine production compared to well-known producers like *H. elongate* DSM 2581^ T^ and *H. salina* DSM 5928^ T^ have been used to synthesize this naturally occurring compound (Yu et al. 2022; Zhang et al. 2022a, b, c). In this study, improved ectoine production was attempted using conventional UV mutagenesis methods. Eight rounds of mutations were used for altering *H. campaniensis* strain XH26 following different exposure times. Stable mutant producer strains were ultimately obtained, and ectoine content facilitated by batch fermentation of these strains was greatly improved. Finally, whole genomic sequencing of the wild-type strain XH26 and mutant strain G_8_-52 was conducted and genomic differences were evaluated in context of ectoine production differences.

## Materials and methods

### Bacterial strains and incubation media

The wild-type *H. campaniensis* strain XH26 (CCTCC^M^2019776) investigated in this study was isolated from the Xiaochaidan Salt Lake in the Chaidamu Basin of China. Culture medium for strain activation (CMSA, w/v) was produced from Oesterhelt-Stoeckenius’s medium (Oesterhelt and Stoeckenius 1974) and contained 5% NaCl, 0.97% MgSO_4_, 0.02% CaCl_2_, 0.2% KCl, 0.3% citric acid sodium, 1% bacterial peptone, and 0.2% yeast extract. Medium pH was adjusted to 7.5 using 3 M NaOH. Culture medium for ectoine accumulation (CMEA, in liter) was produced using an optimized medium containing 8.7% NaCl, 1.2% MgSO_4_, 1.8% KCl, 0.5% sodium l-glutamate, and 1.25% casein enzymatic hydrolysate (Solarbio Life Science, China). Ectoine fermentation was conducted at 35 °C and with media adjusted to pH 8.0.

### Colony morphology and electron microscopy analysis

The colony morphologies of wild-type or mutant strains were investigated on solid CMSA medium after 12 h of growth at 35 °C. Bacterial cells cultured on CMSA media were harvested by centrifugation at 8000 rpm for 5 min (OD_600_ value of approximately 1.20) and suspended in freshly prepared fixative comprising 2.5% glutaraldehyde for 12 h at 4 °C. Samples were then dehydrated in a series of ethanol solutions comprising 30%, 50%, 70%, 80%, 90%, and 100% ethanol (v v^−1^) for 15 min at each concentration. Samples were subsequently centrifuged at 8000 rpm for 1 min and then washed twice in isoamyl acetate for 20 min each wash, followed by centrifugation at 5000 rpm for 3 min. Cell sediments were then frozen at − 20 °C, − 40 °C, and − 80 °C for 6 h and subsequently freeze-dried at − 65 °C for 12 h. The dehydrated samples were sputter-coated with gold using a Hitachi E-1045 coater (Hitachi High-Tech Science Corp., Japan) and examined with a JSM-6610 (JEOL Ltd. Japan) scanning electron microscope (SEM). The SEM acceleration voltage was 15 kV and the EDS working distance was set to 12 mm, while the data acquisition time was set to 600 s, with a speed of 2000 cps.

### HPLC detection of ectoine

Wild-type or mutant strains were activated to grow in liquid CMEA medium for 12 h. Cultures were subsequently placed in 250-mL conical flasks (inocula of 1%, v v^−1^) to grow for over 30 h. A total of 1.5 mL of fermentation liquor was harvested by centrifugation at 8000 rpm for 5 min. The pellets were then resuspended in ethanol (90%, v v^−1^) with rigorous shaking for 2 min, and then ground for 5 min with a high-speed tissue grinder (OSE-Y50, Tiangen Ltd., China). The ethanol extract was centrifuged at 12,000 rpm for 5 min and the supernatant was filtered through a 0.45-mm filter. Ectoine extract concentrations were determined by HPLC analysis using an Aligent Technologies HPLC (1260 Series, America) system with a Merck-SeQuant ZIC-HILIC chromatographic column (150 × 4.6 mm, 5 μm, Germany). Chromatography was performed at a flow rate of 1 mL/min with acetonitrile/ultrapure water (4/1, v v^−1^) as the mobile phase at 30 °C, and detection amount of 10 µL. Ectoine was measured at 210 nm using a UV/VIS detector (Zhu et al. 2014). Standard ectoine (purity greater than 95%) was purchased from Fluka Analytical (Germany) for comparison.

### Multiple rounds of ultraviolet radiation mutagenesis

**Fig. 1:**
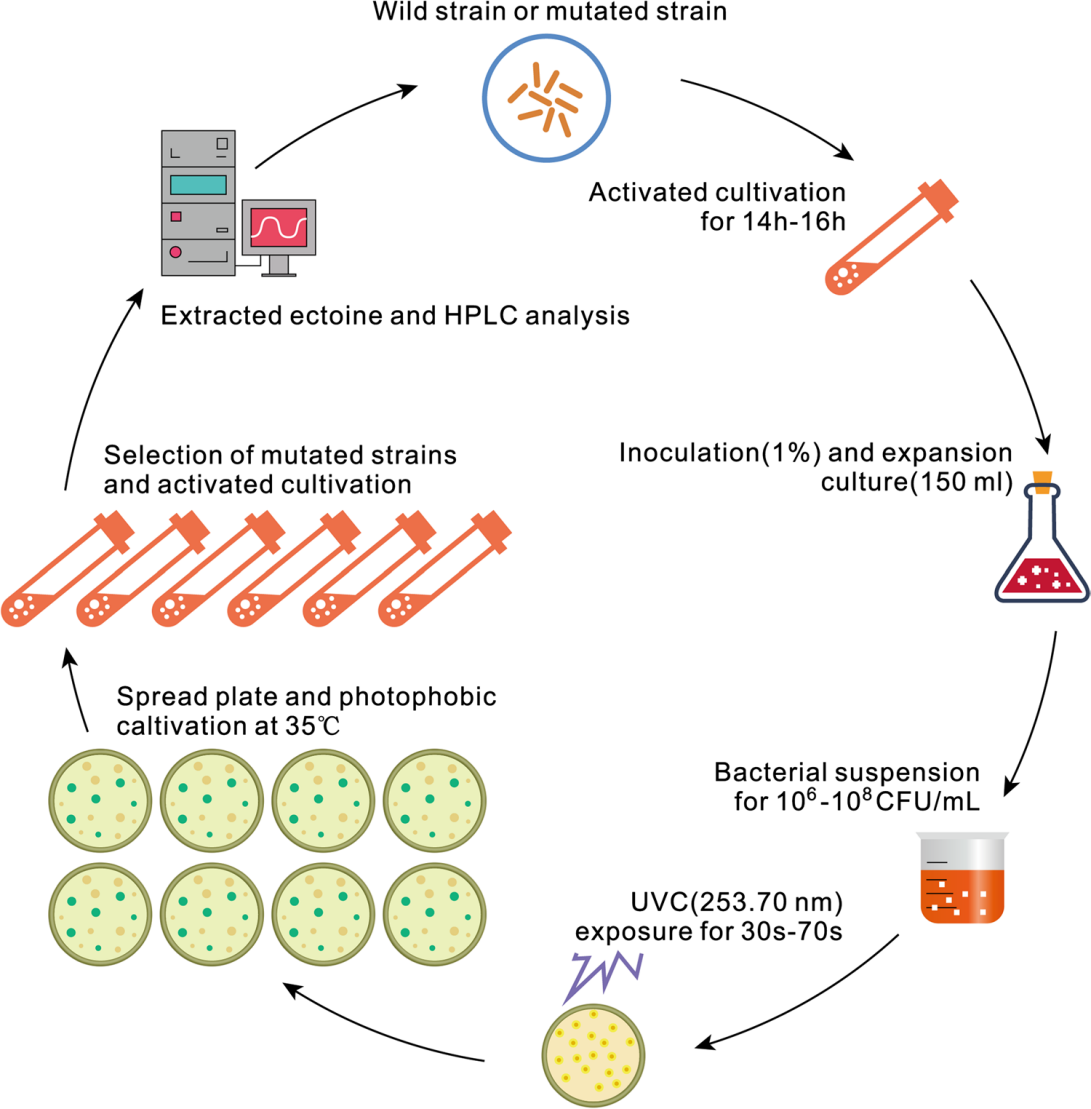
Schematic showing the experimental design with multiple rounds of ultraviolet mutagenesis for the wild-type *H. campaniensis* strain XH26

Wild-type strain XH26 was activated and grown in 150 mL of CMSA medium for 14 to 16 h. Cultures were then diluted with 0.9% NaCl to achieve bacterial cell concentrations of 10^6^ to 10^8^ CFU/mL (Fig. 1). Bacterial suspensions (20 mL) were then distributed on a sterile glass plate (90-mm diameter) and induced using a UV-C lamp at a wavelength of 253.7 nm (220 V, 25 W, ZHJH-C1109C, Shaihai Zhicheng). The distance between the glass plate and the UV lamp was adjusted to 30 cm (Tan et al. 2021). Exposure times of 30, 40, 50, 60, and 70 s were evaluated. Next, 50 µL of bacterial suspensions mutagenized for different times were spread on CMEA agar plates, with duplicates used for each time-point. The plates were incubated for 24 h at 35 °C in the dark to prevent photoreactivation. Mutant colonies were visually identified based on their sizes and growth rates. Colonies were subsequently transferred to liquid CMEA medium. Mutants were then isolated based on their ectoine content production and biomass productivity. Eight rounds of mutagenesis were conducted using the above procedure. Mutants with the most stable mutations were then isolated by in vitro serial sub-culturing for 40 days.

### Determination of cellular abundances and ectoine concentrations

The wild-type XH26 strain and selected mutated strains were inoculated in 250 mL of CMEA medium and cultivated in a rotary shaker at 35 °C with shaking at 120 rpm. A UV–VIS spectrophotometer (SP-754, Shanghai, China) was used to periodically determine cellular biomass growth by measuring OD_600_ absorbances. Intracellular ectoine from the mutated strains was extracted using 90% (v v^−1^) ethanol and then subjected to isocratic HPLC analysis to facilitate ectoine quantification. Intracellular ectoine concentrations were then calculated as milligrams per fermentation broth (L) or cellular dry weight (g).

### Genomic sequencing, assembly, and annotation

High-quality genomic DNAs were extracted from the wild-type strain XH26 and the mutated strain G_8_-52 using the NEBNext®Ultra™ DNA Library Prep Kit for Illumina (New England Biolabs, USA), followed by quantification with a Qubit instrument (v3.0, Thermo Fisher Scientific, USA). Genomic DNAs were then sequenced on a PacBio RS II sequencer at Frasergen Biosciences (Shanghai, China). Genomic sequence assembly was performed using the Hierarchical Genome Assembly Process software program (v.4.0; Dyomin et al. 2019). The Glimmer program (v.3.02; Sengupta and Azad 2023) was used to predict genes, while genes encoding tRNAs and rRNAs were predicted using the tRNAscan-SE (v2.0; Chan et al. 2021), and RNAmmer (v1.2; Baba et al. 2021) software programs, respectively. Other RNA types, including miRNAs, sRNAs, and snRNAs, were predicted using the Infernal software program (v.1.1.2; Singh et al. 2022). The Diamond program (v.0.9.12.113; Buchfink et al. 2015) was also used to annotate genes and predict proteins, including by comparison against the Kyoto Encyclopeida of Genes and Genomes, Clusters of Orthologous Groups, Gene Ontology, Carbohydrate Active Enzymes, and Non-Redundant Protein Sequence databases.

### Comparative genomic analyses

The Mummer software program (v.3.23; Yoon et al. 2017) was used to align the genomes of strains XH26 and G_8_-52, in addition to determining the relative direction of sequences and adjust the genomic alignments. The LastZ software program (v.1.02.00; Liu et al. 2020) was used for whole-genome comparisons. The alignment blocks corresponding to translocations and inversions were identified based on sequences and the relative orientation of the new alignment blocks. Further, structural variation regions between the alignment blocks were determined based on distance relationships between adjacent alignment blocks on the two genomes. The two genomes were also compared against the NCBI database using the basic local alignment search tool (BLAST).

### Statistical analyses

Results were presented as means for three triplicate measurements with error bars showing standard deviations (means ± SD, *n* = 3). Statistical significance was evaluated using analysis of via *t*-tests (SPSS software v. 22.0, IBM Corp., NY) followed by the least significant difference test at 0.05 level (Zhu et al. 2014).

## Results

### Identifying UV-mutated strains

The wild-type strain XH26 was sensitive to UV irradiation. No cell survival was observed following extended exposure to radiation (> 65 s). The cell fatality rate (%) was 95–98% after a UV irradiation time of 50 s to 60 s (data not shown). Screening of eight rounds of mutations associated with ectoine accumulation was conducted (Table 1). The mutated strains were exposed to different induction times ranging from 30 to 60 s and all strains were fast-growing at 35 °C and isolated from CMEA agar plates. However, some UV-mutated cells exhibited higher ectoine content than others. Consequently, the high-yielding representative strains were selected for subsequent mutagenesis. A representative strain after each round of UV mutation exposure was selected from various mutants, thereby leading to optimized ectoine accumulation. Among the 53 colonies generated from the 8th round of mutations, the mutated strains G_8_-52, G_8_-44, and G_8_-17 were isolated because they exhibited the highest ectoine levels of 1.51 ± 0.01 g L^−1^, 1.47 ± 0.02 g L^−1^, and 1.45 ± 0.02 g L^−1^, respectively. The ectoine yield of strain G_8_-52 was surprisingly high compared to that of the original strain XH26 (0.51 ± 0.01 g L^−1^ in optimized CMEA medium), increasing by approximately 200%. Hence, strain G_8_-52, the mutant exhibiting the highest levels of ectoine, was selected for further stability studies to establish its suitability for fermentation applications and subsequent production.

**Table 1:** Ectoine accumulation analysis of wild-type strain and UV-mutated strains for multiple rounds

Mutation rounds	Initiating strain	Sum of mutated strains	Mutated time (s)	Representative strains	Ectoine yield (g/L)	Ectoine yield range of mutated strains (g/L)
0	Wild-type strain XH26	–	–	Strain XH26	0.26 ± 0.02^a^	–
		–	–	Strain XH26	0.51 ± 0.01^b^	–
1	Wild-type strain XH26	18	60	Mutated strain G_1_-8	0.66 ± 0.01	0.38–0.66
			60	Mutated strain G_1_-11	0.60 ± 0.02	
			60	Mutated strain G_1_-9	0.59 ± 0.01	
2	Mutated strain G_1_-8	22	50	Mutated strain G_2_-7	0.77 ± 0.02	0.47–0.77
			50	Mutated strain G_2_-5	0.74 ± 0.01	
			60	Mutated strain G_2_-13	0.69 ± 0.03	
3	Mutated strain G_2_-7	41	50	Mutated strain G_3_-28	0.91 ± 0.02	0.45–0.92
			30	Mutated strain G_3_-13	0.85 ± 0.01	
			60	Mutated strain G_3_-32	0.82 ± 0.01	
4	Mutated strain G_3_-28	38	30	Mutated strain G_4_-17	0.98 ± 0.01	0.44–0.98
			30	Mutated strain G_4_-15	0.84 ± 0.02	
			30	Mutated strain G_4_-9	0.80 ± 0.03	
5	Mutated strain G_4_-17	26	30	Mutated strain G_5_-1	1.03 ± 0.01	0.44–1.03
			30	Mutated strain G_5_-14	0.93 ± 0.03	
			30	Mutated strain G_5_-16	0.93 ± 0.01	
6	Mutated strain G_5_-1	53	30	Mutated strain G_6_-15	1.19 ± 0.01	0.47–1.20
			50	Mutated strain G_6_-11	1.04 ± 0.02	
			30	Mutated strain G_6_-30	0.94 ± 0.03	
7	Mutated strain G_6_-15	63	50	Mutated strain G_7_-37	1.21 ± 0.01	0.40–1.21
			40	Mutated strain G_7_-20	1.19 ± 0.02	
			50	Mutated strain G_7_-43	1.13 ± 0.01	
8	Mutated strain G_7_-37	71	60	Mutated strain G_8_-52	1.51 ± 0.01	0.47–1.51
			50	Mutated strain G_8_-44	1.47 ± 0.02	
			50	Mutated strain G_8_-17	1.45 ± 0.02	

^a^Wild-type strain XH26 was cultivated in CMSA medium

^b^Wild-type strain XH26 and all mutated strains were cultivated in CMEA medium

Calculated mean is for triplicate measurements from two independent experiments

^a–^^b^Means with different superscripts in the same column are considered statistically different (*t*-test, *P* ≤ 0.05)

### Morphology of the wild-type strain XH26 and the mutated strain

**Fig. 2:**
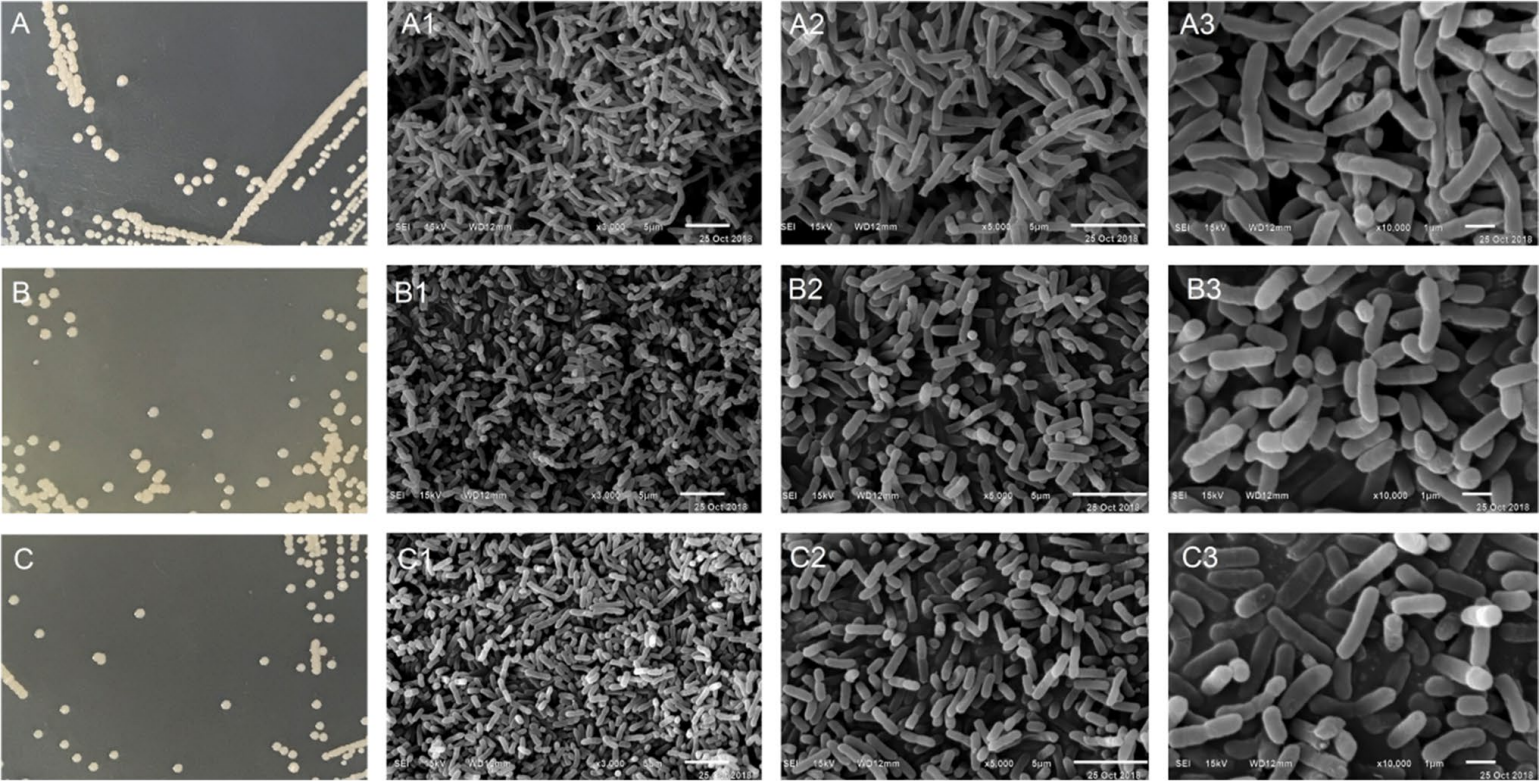
Images showing colony morphology and scanning electron micrographs of wild-type strain XH26 and mutated strains. (A) Wild-type strain colony images. (A1–A3) Scanning electron micrographs of the wild-type strain at 3,000 × , 5,000 × , and 10,000 × magnification, respectively. (B, C) Colony morphologies of the mutated strains G_8_-52 and strain G_8_-44, respectively. (B1–B3, C1–C3) Scanning electron micrographs of G_8_-52 and G_8_-44 cells, respectively. Bars in the 3,000 × , 5,000 × , and 10,000 × microscopic images represent 5 μm, 5 μm, and 1 μm, respectively

The wild-type strain XH26 tolerated high salt concentrations (0 to 3.0 M NaCl), with an optimum salinity of 1.50 M NaCl for growth. This isolate also grew over a pH range of 6.0 to 10.0, with a pH optimum of 8.0. After 12 to 16 h of incubation at 35 °C, milky colonies appeared on CMSA agar plates. The mutated strains G_8_-52 and G_8_-44 were round and small, with a diameter of 1.2 mm (Fig. 2(A, B, C)). The colonies also exhibited adhesion, swelling, moistness, and smooth edges that were non-transparent. Electron microscopy analysis of cellular morphologies revealed that the wild-type XH26 strain and the UV-mutated strains were long rod-shaped cells that were motile and non-flagellated. Wild-type strain XH26 cells were 3 to 5.0 μm in length and 0.5 to 0.75 μm in width (Fig. 2(A1–A3)), while the UV-mutated strain G_8_-52 cells were 1.25 to 3.75 μm in length, with identical widths (Fig. 2(C1–C3)). Mutated strain cells were clearly shorter than the wild-type strain cells, possibly due to UV-induced morphological changes after mutation.

### Growth and ectoine accumulation in the wild-type strain XH26 and the mutant strain G_8_-52

**Fig. 3:**
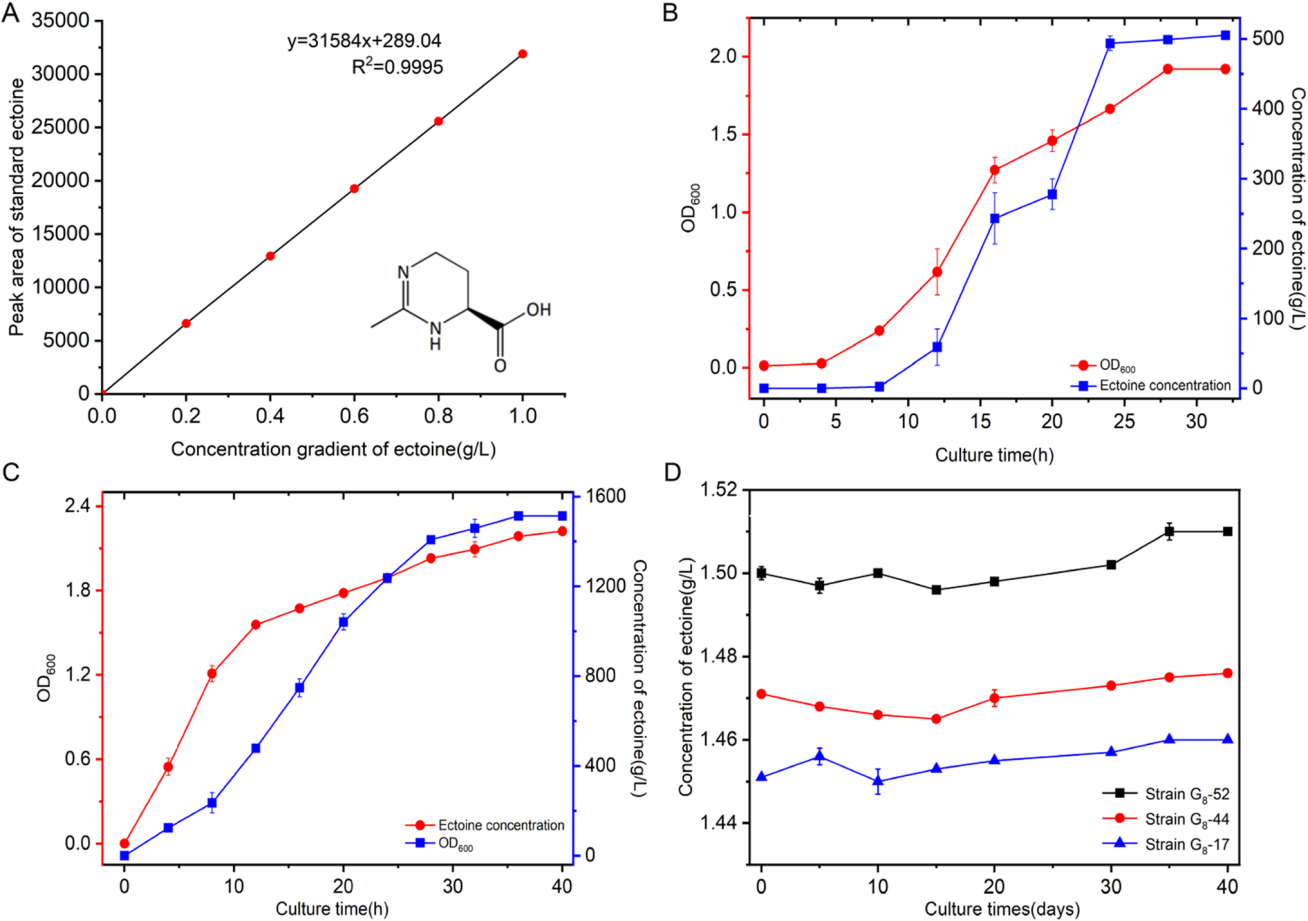
Growth curve and ectoine yield characteristics of the wild-type strain XH26 and the mutant strain G_8_-52. **A** Ectoine standard curve based on HPLC analysis. **B** Ectoine accumulation by the wild-type strain XH26 in CMEA medium. HPLC was performed as follows: mobile phase of acetonitrile/water (4/1, v/v), detection wavelength of 210 nm, flow rate of 1.0 mL/min, column pressure of 3.48–4.76 MPa, column temperature of 30 °C, and detection amount of 10 µL. **C** Ectoine accumulation by the mutated strain G_8_-52. **D** Growth of the mutated strain G_8_-52 and subculture generations of mutated strains in CMEA medium. All strains were cultured in CMEA medium on a rotary shaker at 35 °C and with shaking at 120 rpm. Data represent means of triplicate values with error bars showing standard deviation

HPLC analyses were used to establish a standard curve relationship between peak areas and ectoine concentrations (Fig. 3A). These data were then used for subsequent ectoine measurements. The wild-type strain XH26 accumulated intracellular ectoine to resist osmotic stress in hyper-osmotic environments up to concentrations of 0.26 ± 0.02 g L^−1^ (*p* < 0.05) within primitive CMSA medium. However, optimization of fermentation conditions in CMEA medium at pH 8.0 and incubation at 35 °C led to significantly higher ectoine accumulation of 0.51 ± 0.01 g L^−1^ (*p* < 0.01). Cell densities (OD_600_) and ectoine concentrations increased with increased culture time (Fig. 3B), indicating that culture times > 24 h were conducive for ectoine production. The encouraging results observed for mutant strain G_8_-52 in terms of cell biomass and ectoine concentration levels led to the implementation of continuous culture conditions in CMEA medium. The mutant strain G_8_-52 grew faster (OD_600_ value of 1.20 after 8 h) compared with the wild-type strain XH26 (OD_600_ value of 0.25 after 8 h) (Fig. 3C). Further, ectoine concentration analysis revealed an S-type accumulation curve for strain G_8_-52 during the rapid growth stage from 8 to 28 h and in the steady accumulation phase between 28 and 40 h. The mutated strain G_8_-52 was then grown continuously for 40 days in CMEA medium to confirm ectoine production (Fig. 3D). Ectoine content remained unchanged at 1.50 g L^−1^ (0.65 g g^−1^ of ectoine/cell dry weight).

### Genomic characteristics of the wild-type strain XH26 and the mutant strain G_8_-52

Genomic features of the wild-type strain XH26 and the mutant strain G_8_-52 were evaluated (Table S1). The complete genomes of the wild-type strain XH26 and mutant strain G_8_-52 were assembled in circular contigs of size 4,112,053 and 4,098,386 bp, respectively, and contained similar GC contents of 52.62% and 52.53%, respectively. The wild-type strain XH26 genome encoded 3927 predicted genes including 3832 protein-coding, 63 tRNA, and 32 rRNA genes. Similarly, the mutant strain G_8_-52 genome encoded 3945 predicted genes including 3849 protein-coding, 64 tRNA, and 32 rRNA genes. Additional summary statistics and functional categorization of genes based on comparison to multiple functional databases are shown in supplementary Fig. S2 for wild-type strain XH26 and Fig. S3 for mutant strain G_8_-52.

### Genome comparisons between wild-type strain XH26 and mutant strain G_8_-52

Whole genome sequencing revealed the presence of 24 mutant sites including ten nucleotide insertions, ten nucleotide deletions, and four unique single nucleotide polymorphisms (Table 2 and Table S2). BLAST analysis revealed two nonsense mutations (orf00034 and orf03151) in non-coding genetic regions. The other 22 mutations occurred in coding regions including in orf00215 (DNA-directed RNA polymerase subunit), orf00258 (DUF6164 family protein), orf00263 (hypothetical protein), orf00443 (membrane dipeptidase), orf00721 (AraC family transcriptional regulator), orf00723 (ornithine cyclodeaminase), orf00726 (TRAP transporter large permease), orf02141 (branched-chain amino acid ABC transporter), orf02186 (energy transducer TonB), orf02266 (cell division protein ZapE), orf02522 (membrane protein), orf03335 (molybdopterin molybdenumtransferase MoeA), orf03412 (5-dehydro-2-deoxygluconokinase), orf03417 (MurR/RpiR family transcriptional regulator), orf03539 (quinohemoprotein amine dehydrogenase), orf03568 (indolepyruvate ferredoxin oxidoreductase family protein), orf03677 (manganese transporter), orf00424 (hypothetical protein), orf02403 (β-3-deoxy-d-manno-oct-2-ulosonic acid), orf02552 (secretion protein HylD), and orf03427 (GntR family transcriptional regulator). Notably, *orf00723* encodes ornithine cyclodeaminase and the gene *lipA* encoding capsular polysaccharide biosynthesis proteins had mutated into *davT* that encodes a 4-aminobutyrate transaminase (NCBI identity of 99.76%) and the gene *gabD* that encodes a NAD-dependent succinate-semialdehyde dehydrogenase (NCBI identity of 99.38%) that are both involved in the glutamate metabolism pathway of succinic semialdehyde and succinic acid (Fig. S3).

**Table 2:** Analysis of mutation sites of wild strain XH26 and mutant strain G_8_-52

No	ORF numbers	Reference gene of XH26	Reference proteins of wild strain XH26 (NCBI identity%)	NCBI accession no	Alter Gene of G_8_-52	Alter proteins of mutant strain G_8_-52 (NCBI identity%)	NCBI accession no
1	orf00034	*orf00034*	RNA-binding protein S1 (100%)	WP030071723.1	*cvfB*	RNA-binding protein S1 (100%)	WP038480019.1
2	orf00215	*rpoB*	DNA-directed RNA polymerase subunit (100%)	WP038480365.1	*thiE*	Thiamine phosphate synthase (100%)	WP038480426.1
3	orf00258	*orf00258*	DUF6164 family protein (96.77%)	WP083000476.1	*orf00272*	Hypothetical protein (82.61%)	BCB06348.1
4	orf00263	*orf00263*	Hypothetical protein (100%)	WP038480446.1	*ugpB*	Glycerol-3-phosphate ABC transporter substrate-binding protein (100%)	WP038480516.1
5	orf00263	*orf00263*	Hypothetical protein (100%)	WP038480446.1	*ugpB*	Glycerol-3-phosphate ABC transporter substrate-binding protein (100%)	WP038480516.1
6	orf00443	*orf0043*	Membrane dipeptidase (100%)	WP038480829.1	*todJ*	2-Keto-4-pentenoate hydratase (99.63%)	AIA74849.1
7	orf00721	*lumQ*	AraC family transcriptional regulator (100%)	WP038481501.1	*dinB*	DNA polymerase IV (100%)	WP095602669.1
8	orf00723	*orf00723*	Ornithine cyclodeaminase (99.04%)	WP095602694.1	*davT*	4-Aminobutyrate-2-oxoglutarate transaminase (99.76%)	WP120385708.1
9	orf00726	*siaT*	TRAP transporter large permease (98.92%)	WP009722136.1	*orf00732*	CoA transferase (100%)	WP038481597.1
10	orf02141	*orf02141*	Branched-chain amino acid ABC transporter (100%)	WP038485114.1	*panE*	Ketopantoate reductase family protein (95.10%)	WP231657074.1
11	orf02186	*tonB*	Energy transducer TonB (100%)	WP095603125.1	*aroF*	3-Deoxy-7-phosphoheptulonate synthase (100%)	WP038485201.1
12	orf02266	*orf02266*	Hypothetical protein (77.27%)	EHJ91333.1	*lpoA*	Penicillin-binding protein activator (100%)	WP095603166.1
13	orf02522	*orf02522*	Membrane protein (100%)	WP038486986.1	*ycjM*	Sugar phosphorylase (100%)	WP038486080.1
14	orf03151	*orf03151*	Hypothetical protein (79.86%)	WP015488478.1	*orf03151*	Hypothetical protein (92.45%)	MCB1853323.1
15	orf03335	*moeA*	Molybdopterin molybdenumtransferase MoeA (99.52%)	WP198349446.1	*moeA*	3,4-Dihydroxy-2-butanone 4-phosphate synthase (67.65%)	CDT26995.1
16	orf03412	*iolC*	5-Dehydro-2-deoxygluconokinase (100%)	WP038478787.1	*cysA2*	2-Aminoethylphosphonate ABC transporter ATP-binding protein (100%)	WP038478825.1
17	orf03417	*glk*	MurR/RpiR family transcriptional regulator (100%)	WP038478801.1	*orf03444*	EAL domain-containing protein (99.78%)	WP038478837.1
18	orf03539	*qhnDH*	Quinohemoprotein amine dehydrogenase (100%)	WP051626386.1	*paaA*	1,2-Phenylacetyl-CoA epoxidase subunit A (100%)	WP198348883.1
19	orf03568	*iorA*	Indolepyruvate ferredoxin oxidoreductase family protein (99.40%)	WP231657858.1	*orf03597*	YihY/virulence factor BrkB family protein (99.68%)	WP038479174.1
20	orf03677	*mntA*	Zinc ABC transporter substrate-binding protein (96.56%)	WP231657799.1	*citT*	Response regulator (100%)	WP095603464.1
21	orf00424	*orf00424*	Hypothetical protein (68.57%)	RAZ03907.1	*cntA*	Aromatic ring-hydroxylating dioxygenase subunit alpha (99.53%)	WP095604661.1
22	orf02403	*lipA*	Capsular polysaccharide biosynthesis protein (97.50%)	WP095603249.1	*gabD*	NAD-dependent succinate-semialdehyde dehydrogenase (99.38%)	WP038485827.1
23	orf02552	*apxIB*	Type I secretion system permease/ATPase (99.72%)	WP038476878.1	*bepC*	TolC family outer membrane protein (99.39%)	WP198350197.1
24	orf03427	*phnR*	GntR family transcriptional regulator (100%)	WP038478831.1	*katG*	Catalase/peroxidase HPI (99.86%)	WP038478864.1

## Discussion

Previous studies have indicated strategies that can improve the ability of strains to produce ectoine. For example, these strategies include using recombinant producer strains involving the synthesis of *ectABC* or *ask*-*ectABCD* gene clusters (Stöveken et al. 2011; He et al. 2015; Zhang et al. 2022a, b, c), defective mutant strains with altered *teaABC* and *araC* (Fatollahi et al. 2021; Zhang et al. 2022a, b, c), and reconstructed strains based on metabolic engineering (Ma et al. 2022; Zhang et al. 2022a, b, c). Meanwhile, some studies have indicated that intracellular ectoine levels in *Halomonas* strains are highly regulated by external salt levels, medium composition, and culture conditions (Liu et al. 2021; Zhang et al. 2022a, b, c). For example, strain growth and ectoine synthesis were better when glutamate was used as the carbon and nitrogen source (Hobmeier et al. 2022). In this study, CMEA medium containing sodium l-glutamate promoted the ectoine synthesis pathway and the reactions associated with the transformation of oxaloacetic acid into aspartic acid and aspartic acid-*β*-semialdehyde into l-2, 4-diaminobutyric acid. These results were similar to those previously observed following batch fermentation in MG medium (320 mM of monosodium glutamate and 0.5 M NaCl) with *H. salina* DSM 5928^ T^ (Liu et al. 2021; Yu et al. 2022).

To generate a stable high-yielding strain, UV-mutation techniques were used here to conduct multiple rounds of mutations. The main principle of ultraviolet mutation is to make two adjacent thymines form aggregates between double strands of DNA or on the same strand, which hinders the separation and replication of double strands and the normal pairing of bases, thus causing mutation. It should be noted that UV radiation also confers genetically and physiologically deleterious effects leading to metabolic changes within microorganisms (Guihéneuf et al. 2010). Gamma rays, as the highest energy ionizing radiation, can cause mutations of DNA double-stranded or single-stranded breaks in many ways, including structural changes or deletions, oxidation of bases and base sites, and DNA–protein cross-linking (Gaddini et al. 2023). El-Sayed et al. (2019) used gamma and UV irradiation mutagenesis to improve the paclitaxel production of *Aspergillus fumigatus* from 414.32 to 495.31 μg L^−1^. Similarly, UV radiation was used for production enhancement of mycophenolic acid by *Penicillium chrysosporium* (Ribeiro et al. 2011). Moreover, several reports suggested that ultraviolet and gamma ray irradiation as a powerful tool to improve microbial strains by inducing microbial cell mutation (Ismaiel et al. 2014). This strategy resulted in the generation of the mutated *H. campaniensis* strain G_8_-52 that produced 1.51 ± 0.01 g L^−1^ ectoine in shake-flask culture. Strain G_8_-52 exhibited obvious advantages compared with wild-type strain XH26 and other representative *Halomonas* strains (Table S3) in terms of cellular biomass, growth rates (OD_600_ value of 1.20 within 8 h), and the presence of stable genetic traits. However, this is the first study to reveal improved ectoine content in wild-type and associated UV-mutated strains. UV mutagenesis or repeated multiple rounds of UV mutagenesis strategies can provide new insights into how to improve ectoine production.

Ectoine biosynthesis is regulated by the enzymes l-2,4-aminobenzoic acid N_γ_-acetyltransferase, l-alanine transaminase, and ectoine synthetase that are encoded by *ectA*, *ectB*, and *ectC*, respectively (Dutta and Bandopadhyay 2022). Ectoine biosynthesis is also closely related to the l-aspartic acid (oraspartic acid-*β*-semialdehyde) pathway, upstream amino acid metabolism networks (e.g., asparagine, glutamate, and glutamine pathways), and the tricarboxylic acid (TCA) cycle (e.g., through succinic, fumaric, and oxaloacetic acids; Fig. S3). l-Aspartic acid that is involved in ectoine metabolism is synthesized from oxaloacetic acid that is a key intermediate in the TCA cycle. 4-Aminobutyrate transaminase (expressed from the mutant gene *davT*) and NAD-dependent succinate-semialdehyde dehydrogenase (expressed from the mutant gene *gabD*) enhance the conversion of glutamate to succinic semialdehyde or succinic acid, resulting in greater succinic acid that can participate in the TCA cycle and that may then contribute to high metabolic fluxes of oxaloacetic acid, l-aspartic acid, and ectoine (Chen et al. 2022).

Some proteins in the wild-type strain XH26 were notably mutated into ABC transporters (e.g., orf00258 and orf03412, Table 2). ABC transporters are important membrane proteins that mediate the exchange of chemical compounds inside and outside of biofilm and intracellular signals. ABC transporter proteins are widely distributed among various organisms and can transport proteins, amino acids, sugars, and other compounds. In addition, the specific membrane spanning domain of ABC transporters may be involved in bacterial responses to environmental changes (Sylvia et al. 2023; Coumes-Florens et al. 2011) that may accelerate greater transport of metabolic precursor substrates from nutrient media, resulting in greater accumulation of intracellular ectoine (Azarbaijani et al. 2015).

Lastly, the ectoine production of wild-type *H. campaniensis* strain XH26 was improved following multiple rounds of UV mutations and the mutated strain was genetically stable. The mutant strain G_8_-52 generated in this study exhibited the highest cellular growth rates and ectoine yields among numerous generated mutants compared to the wild-type strain. Specifically, total ectoine content produced by the UV mutant significantly increased to 1.51 ± 0.01 g L^−1^ (0.65 g g^−1^ of CDW), representing a two-fold increase compared to the wild-type strain XH26 under the same culture conditions. The mutant strain G_8_-52 also exhibited acceptable stable properties that would render it suitable for use in subsequent fermentation production applications.

### Supplementary Information

Below is the link to the electronic supplementary material.

Supplementary file1 (DOCX 2426 KB)

## Data Availability

The whole genome data could be accessed under accession number SRR19749374 and SRR19749563 in the NCBI database (www.ncbi.nlm.nih.gov).
