# Supplementary material for: Comparative genomic analysis of Halomonas campaniensis wild-type and ultraviolet radiation-mutated strains reveal genomic differences associated with increased ectoine production
Source: Int Microbiol. 2023 Apr 17;26(4):1009–20. doi: 10.1007/s10123-023-00356-y (PMC10622362; doi:10.1007/s10123-023-00356-y)
Supplement: Supplementary file 1 — Supplementary file1 (DOCX 2426 KB) [file 10123_2023_356_MOESM1_ESM.docx]

**Table S1. Genomic features of the wild-type strain XH26 and the mutant strain G_8_-52.**

| **Features** | **Wild-type strain XH26** | **Mutant strain G_8_-52** |
| --- | --- | --- |
| Molecule shape | Circular | Circular |
| Genome size (bp) | 4,112,053 | 4,098,386 |
| GC content (%) | 52.62% | 52.53% |
| Coding sequences (CDS)  Total RNA genes | 104,708  97 | 65,625  98 |
| tRNAs  5S rRNAs  16S rRNAs  23S rRNAs | 63  6  6  6 | 64  6  6  6 |
| rRNA (5S/6S/23S)  Other RNA genes  Coverage (%) | 1/1/1  16  100% | 1/1/1  16  100% |
| Predicted coding genes  Total length of predicted coding genes (bp) | 3,927  3,687,732 | 3,945  3,670,458 |

**Table S2. Genomic comparison of wild-type strain XH26 and mutant strain G_8_-52 regions.**

| No. | ORF Number | Locus | Block | Reference | Change | Type |
| --- | --- | --- | --- | --- | --- | --- |
| 1 | orf00034 | 37964 | exonic | - | G | Insertion |
| 2 | orf00215 | 222002 | exonic | A | - | Deletion |
| 3 | orf00258 | 257737 | upstream | - | C | Insertion |
| 4 | orf00263 | 261715 | upstream | - | T | Insertion |
| 5 | orf00263 | 261744 | upstream | - | AG | Insertion |
| 6 | orf00443 | 436707 | downstream | A | - | Deletion |
| 7 | orf00721 | 727519 | upstream | T | - | Deletion |
| 8 | orf00723 | 729148 | upstream | C | - | Deletion |
| 9 | orf00726 | 732078 | exonic | - | GT | Insertion |
| 10 | orf02141 | 2212367 | exonic | - | C | Insertion |
| 11 | orf02186 | 2262995 | downstream | - | G | Insertion |
| 12 | orf02266 | 2342959 | exonic | T | - | Deletion |
| 13 | orf02522 | 2628488 | exonic | - | C | Insertion |
| 14 | orf03151 | 3296311 | exonic | C | - | Deletion |
| 15 | orf03335 | 3483088 | upstream | G | - | Deletion |
| 16 | orf03412 | 3568183 | exonic | - | T | Insertion |
| 17 | orf03417 | 3574134 | exonic | C | - | Deletion |
| 18 | orf03539 | 3712840 | exonic | C | - | Deletion |
| 19 | orf03568 | 3745920 | exonic | A | - | Deletion |
| 20 | orf03677 | 3867248 | downstream | - | G | Insertion |
| 21 | orf00424 | 419787 | exonic | C | T | Nonsynonymous SNV |
| 22 | orf02403 | 2482248 | exonic | C | T | Synonymous SNV |
| 23 | orf02552 | 2679511 | upstream | A | T | Synonymous SNV |
| 24 | orf03427 | 3584077 | upstream | A | T | Synonymous SNV |

**Table S3. Representative *Halomonas* strains and ectoine production in shake-flask cultures.**

| **Representative strains** | **Type** | **Ectoine yield (g/L or CDW^a^)** | **Culture medium** | ***Ref*.** |
| --- | --- | --- | --- | --- |
| ***H. campaniensis* strain XH26** | Wild | 0.51±0.01 g/L | CMEA | This study |
| ***H. campaniensis*** **strain G_8_-52** | Mutated | 1.51±0.01 g/L (0.65 g/g of CDW) | CMEA | This study |
| ***H. campaniensis* strain G_8_-44** | Mutated | 1.47±0.02 g/L (0.64 g/g of CDW) | CMEA | This study |
| ***H. salina*** | Wild | 0.52±0.02 g/L | CMEA | Our study |
| ***H. elongata*** **strain KS3** | Wild | 0.12 g/g of CDW (2.56 M NaCl) | M63S-3 | Ono et al. 1998 |
| ***H. ventosae* strain DL7** | Wild | 0.46± 0.01 g/L | Nutrient | Zhu et al. 2007  Zhu et al. 2007  Zhu et al. 2007 |
| ***H. ventosae* DSM 15911^T^** | Wild | 0.43± 0.01 g/L | Nutrient |  |
| ***H. halodenitrificans* DSM 735****^T^** | Wild | 0.33± 0.01 g/L | Nutrient |  |
| ***H. boliviensis* strain LC1^T^** | Wild | 0.74 g/L  1.25 g/L | HM  Seed | Guzmán et al. 2007 Van-Thuoc et al. 2010 |
| ***H. salina* DSM 5928^T^** | Wild | 0.29 g/L  0.82 g/L  1.08 ± 0.02 g/L | Nutrient  MM63  MG | Zhu et al. 2007  Zhang et al. 2009  Zhang et al. 2009 |
| ***H. elongata* DSM 2581^T^** | Wild | 0.81± 0.02 g/L | MM63 | Zhang et al. 2009 |
| ***H. elongata* OUT 30018** | Engineered | 0.54 g/g of CDW | MM63 ^b^ | Tanimura et al. 2013 |
| ***Halomonas* sp. strain H02** | Wild | 1.84 g/L | ZMG | Li et al. 2017 |
| ***H. hydrothermalis* strain Y2** | Wild | 0.10 g/g CDW | MG | Zhao et al. 2018 |

^a^CDW: cell dry weight (g/g, ectoine per cell dry weight).

^b^The engineered *H. elongata* strain produced 377 mmol/kg CDW of ectoine from a glucose/xylose mixture.

Calculated mean is for triplicate measurements from two independent experiments ^a-b^ Means with different superscripts in the same column are considered statistically different (*t*-test, *P* ≤ 0.05)


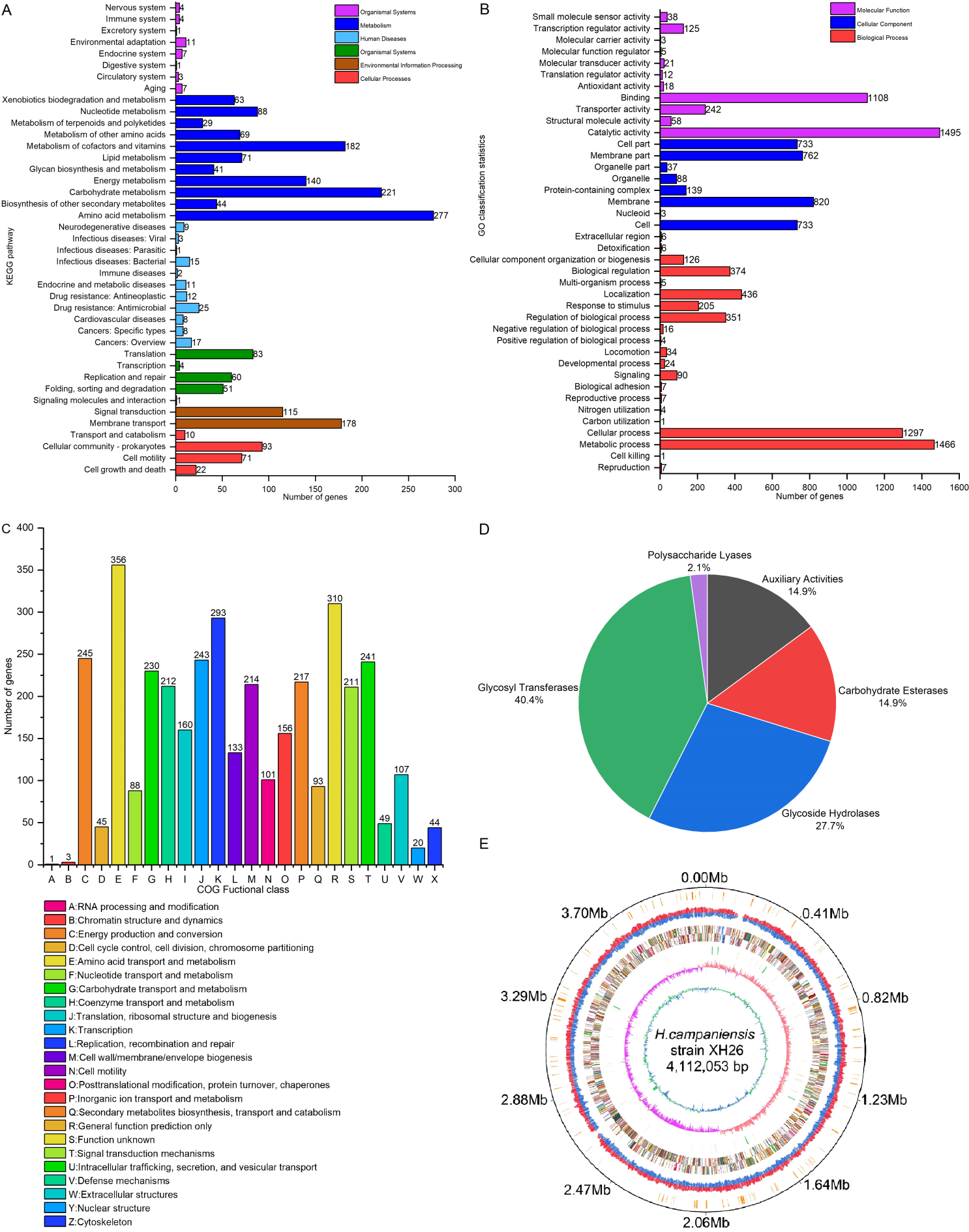


**Fig. S1.** Functional annotations of the wild-type strain XH26. **A)** KEGG annotations, **B**) GO annotations, **C**) COG annotations, **D**) CAZyme annotations, and **E**) Circular chromosome map.


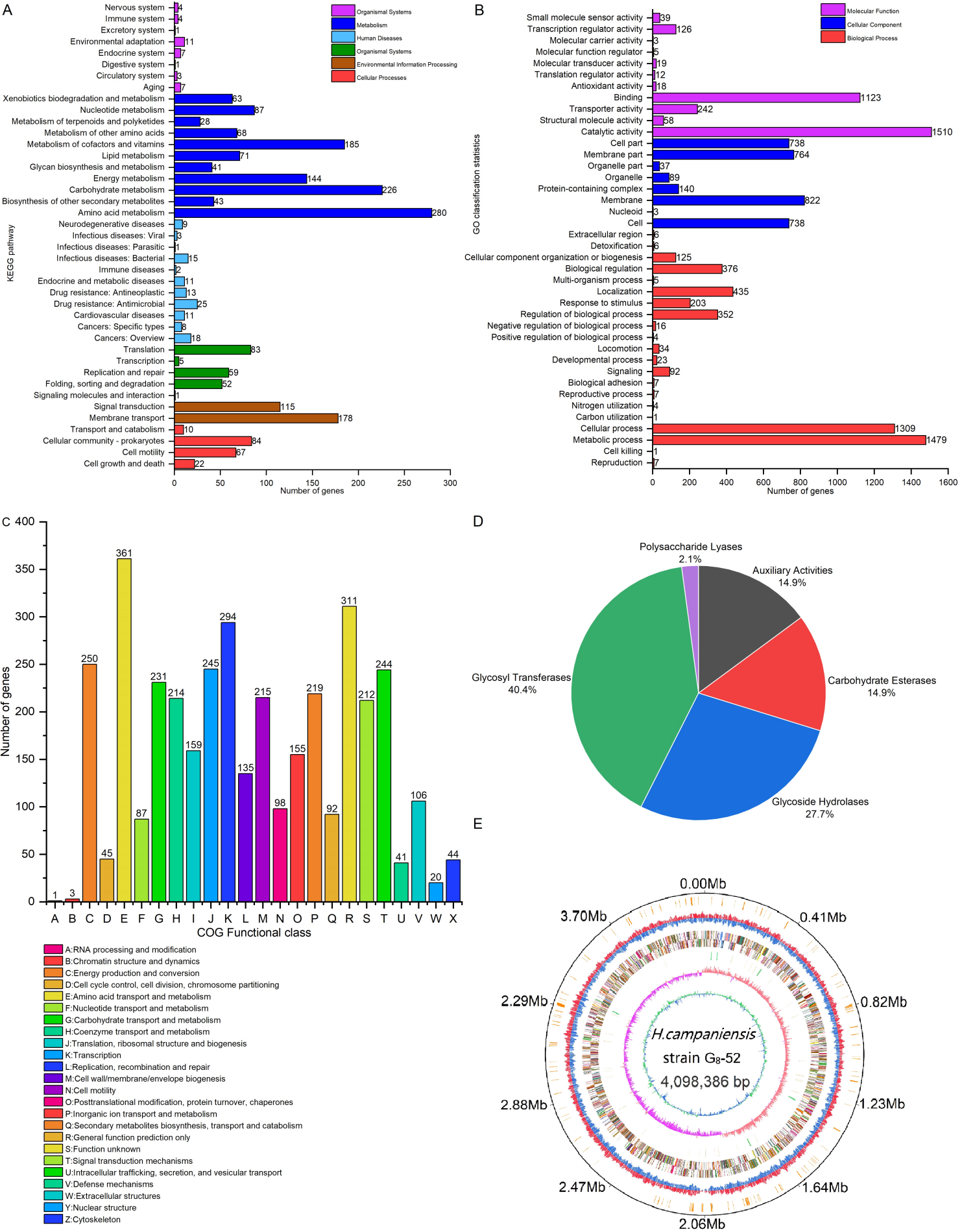


**Fig. S2.** Functional annotations of the mutated strain G_8_-52. **A)** KEGG annotations, **B**) GO annotations, **C**) COG annotations, **D**) CAZyme annotations, and **E**) Circular chromosome map.

**
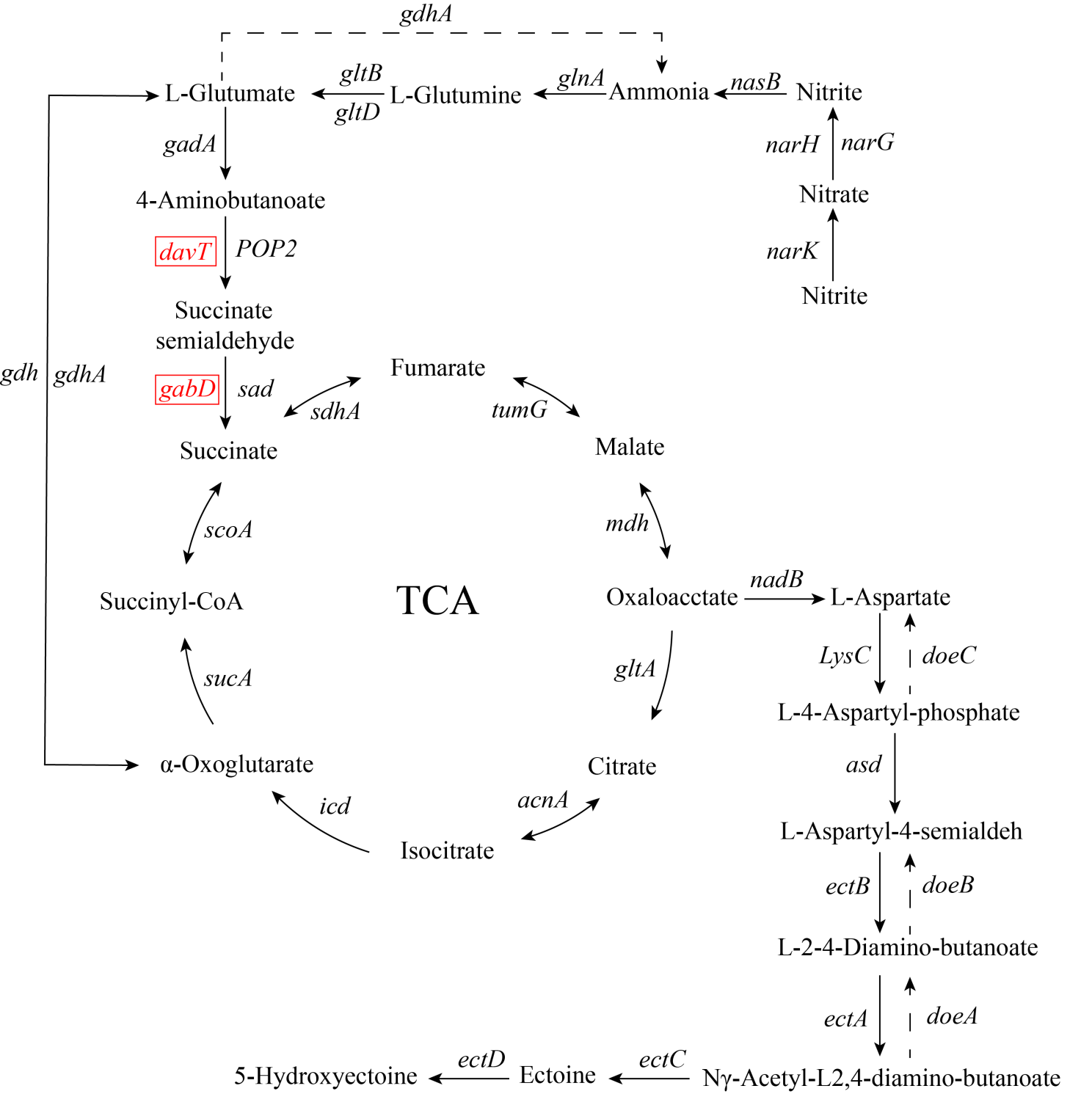
**

**Fig. S3.** The ectoine synthesis pathway and intermediates involved in the tricarboxylic acid cycle of wild strain XH26. TCA is a tricarboxylic acid cycle. Gene *davT* and *gabD* were found to be involved in the tricarboxylic acid cycle, which leads to the increase of ectoine production.
